# Integrated analysis of microRNA and mRNA expression profiling identifies *BAIAP3* as a novel target of dysregulated hsa-miR-1972 in age-related white matter lesions

**DOI:** 10.18632/aging.202562

**Published:** 2021-02-09

**Authors:** Wen-Qing Huang, Qing Lin, Shuai Chen, Lixiang Sun, Qingjie Chen, Kehui Yi, Zhi Li, Qilin Ma, Chi-Meng Tzeng

**Affiliations:** 1 Shanghai Institute of Precision Medicine (SHIPM), Ninth People’s Hospital, Shanghai Jiao Tong University School of Medicine, Shanghai, China; 2 Translational Medicine Research Center (TMRC), School of Pharmaceutical Sciences, Xiamen University, Xiamen, Fujian, China; 3 Department of Neurology, The First Affiliated Hospital of Xiamen University, Xiamen, Fujian, China; 4 Xiamen Key Laboratory of Brain Center, The First Affiliated Hospital of Xiamen University, Xiamen, Fujian, China; 5 School of Medicine, Xiamen University, Xiamen, Fujian, China; 6 The First Clinical College of Fujian Medical University, Fuzhou, Fujian, China; 7 Department of Otolaryngology-Head and Neck Surgery, Xiamen Key Laboratory of Otolaryngology-Head and Neck Surgery, The First Affiliated Hospital of Xiamen University, Xiamen, Fujian, China; 8 Chen Zhi-nan Academician Workstation, Institute of Basic and Translational Medicine, Xi’an Medical University, Xi’an, Shanxi, China; 9 Department of Nuclear Medicine, The First Affiliated Hospital of Nanchang University, Nanchang, Jiangxi, China; 10 Department of Neurology, Zhongshan Xiamen Hospital, Fudan University, Xiamen, Fujian, China; 11 College of Pharmaceutical Sciences, Nanjing Tech University, Nanjing, Jiangsu, China

**Keywords:** white matter lesions (WMLs), leukoaraiosis (LA), biomarkers, microRNA (miRNA), BAIAP3

## Abstract

White matter lesions known as leukoaraiosis (LA) are cerebral white matter hyperintensities observed in elderly individuals. Currently, no reliable molecular biomarkers are available for monitoring their progression over time. To identify biomarkers for the onset and progression of LA, we analyzed whole blood-based, microRNA expression profiles of leukoaraiosis, validated those exhibiting significant microRNA changes in clinical subjects by means of quantitative real-time polymerase chain reactions and determined the function of miRNA in cell lines by means of microRNA mimic transfection assays. A total of seven microRNAs were found to be significantly down-regulated in leukoaraiosis. Among the microRNAs, hsa-miR-1972 was downregulated during the early onset phase of leukoaraiosis, as confirmed in independent patients, and it was found to target leukoaraiosis-dependent *BAIAP3,* decreasing its expression in 293T cell lines. Functional enrichment analysis revealed that significantly dysregulated miRNAs-mRNAs changes associated with the onset of leukoaraiosis were involved in neurogenesis, neuronal development, and differentiation. Taken together, the study identified a set of candidate microRNA biomarkers that may usefully monitor the onset and progression of leukoaraiosis. Given the enrichment of leukoaraiosis-associated microRNAs and mRNAs in neuron part and membrane system, *BAIAP3* could potentially represent a novel target of hsa-miR-1972 in leukoaraiosis through which microRNAs are involved in the pathogenesis of white matter lesions.

## INTRODUCTION

White matter lesions (WMLs), also termed leukoaraiosis (LA) or white matter hyperintensities (WMHs), appear as signal hyperintensities in periventricular and subcortical areas on T2-weighted and fluid-attenuated, inversion recovery, (FLAIR)-magnetic resonance imaging (MRI) brain scans [1]. LA is common in elderly patients and highly correlated with age [2, 3]. Thus, it is often referred to as an age-related white matter lesion [2, 3]. Previous population-based studies have reported that its prevalence ranges from 58.3% to 100% in older patients [3–6].

Pathologically, LA is mainly characterized by demyelination, gliosis, partial loss of axons, and an enlargement of perivascular spaces [1, 3, 7, 8]. These pathological characteristics may be due to incomplete ischemia [3, 7, 8]. Although its symptoms are not clearly evident, LA is considered harmful [9]. Clinically it associates with neurologic disorders, including dementia, Parkinson’s disease, and stroke [8, 10–13]; psychiatric disorders, such as depression and schizophrenia [8, 14, 15]; and inflammatory diseases, such as multiple sclerosis and systemic lupus erythematosus [8, 16, 17]. LA is a complex disease, which is etiologically multifactorial, due to both environmental and genetic factors. Age and hypertension are the chief risk factors for LA [2, 3]. Other risk factors include hypertension, diabetes mellitus, smoking, drinking, and abnormal homocysteine and low-density lipoprotein cholesterol levels [2, 3, 6]. Significant genetic effects are also contributory [18–20]. During the past several decades, numerous risk genes and single nucleotide polymorphisms have been found to increase the risk of LA, supporting a strong genetic component [21–35]. However, its precise pathogenesis is poorly understood to date. Moreover, with the exception of MRI technology, it is challenging to diagnose LA patients with other low-cost methods. Since LA is risk factor for declines in cognitive ability and motor function, and psychiatric health [2, 13, 36–39], it is becoming a substantial public health burden, further aggravated by a growing elderly population. Accordingly, it will be necessary to elucidate its pathogenicity in order to develop efficient strategies for prevention and management.

MicroRNA (miRNA) is a classic category of single-stranded small (20–22 nucleotide) RNA, which can inhibit gene expression at the posttranscriptional level via its binding to the 3’UTR region of target mRNA [40]. It is an important regulator of neuronal proliferation, differentiation, apoptosis, and development [41, 42], and is involved in human disorders including neurodegenerative diseases (e.g., Alzheimer disease and Parkinson disease) [43–45], neuropsychiatric diseases (e.g., schizophrenia and Rett syndrome) [46, 47], autoimmune diseases (e.g., multiple sclerosis and rheumatoid arthritis) [48–50], and cancers (e.g., breast cancer and hepatocellular carcinoma) [51–53]. Significantly, miRNAs are present in a stable form in human whole blood and plasma [54, 55]. The expression levels of miRNAs in plasma could act as biomarkers for disease prognosis [54, 55]. Thus, they could be used both as drug targets or as diagnostic tools.

To date, studies on the genetics of LA have focused on susceptible genes and abnormal gene expression [21, 22, 26–31, 56–58]. Very few studies have been carried out to identify the epigenetic factors involved in regulating transcription of risk genes associated with LA. Therefore, our lab has focused on the determination of abnormal DNA methylation and miRNA expression in LA with an overall objective of elucidating the epigenetic mechanisms of LA pathogenesis [23, 59]. In particular, miRNA profiles have yet to be investigated, and the pathogenetic role of miRNA in LA has not been explored. In this study, we investigated miRNA biomarkers in LA patient plasma using miRNA microarray analysis and also explored the potential functions of aberrant miRNA in LA pathogenesis using molecular biology and bioinformatics protocols. Our study revealed epigenetic involvement in LA pathogenesis and identified candidate biomarkers useful for LA diagnosis.

## RESULTS

### Differentially expressed miRNA implicated in the onset and progression of LA

**Figure 1:**
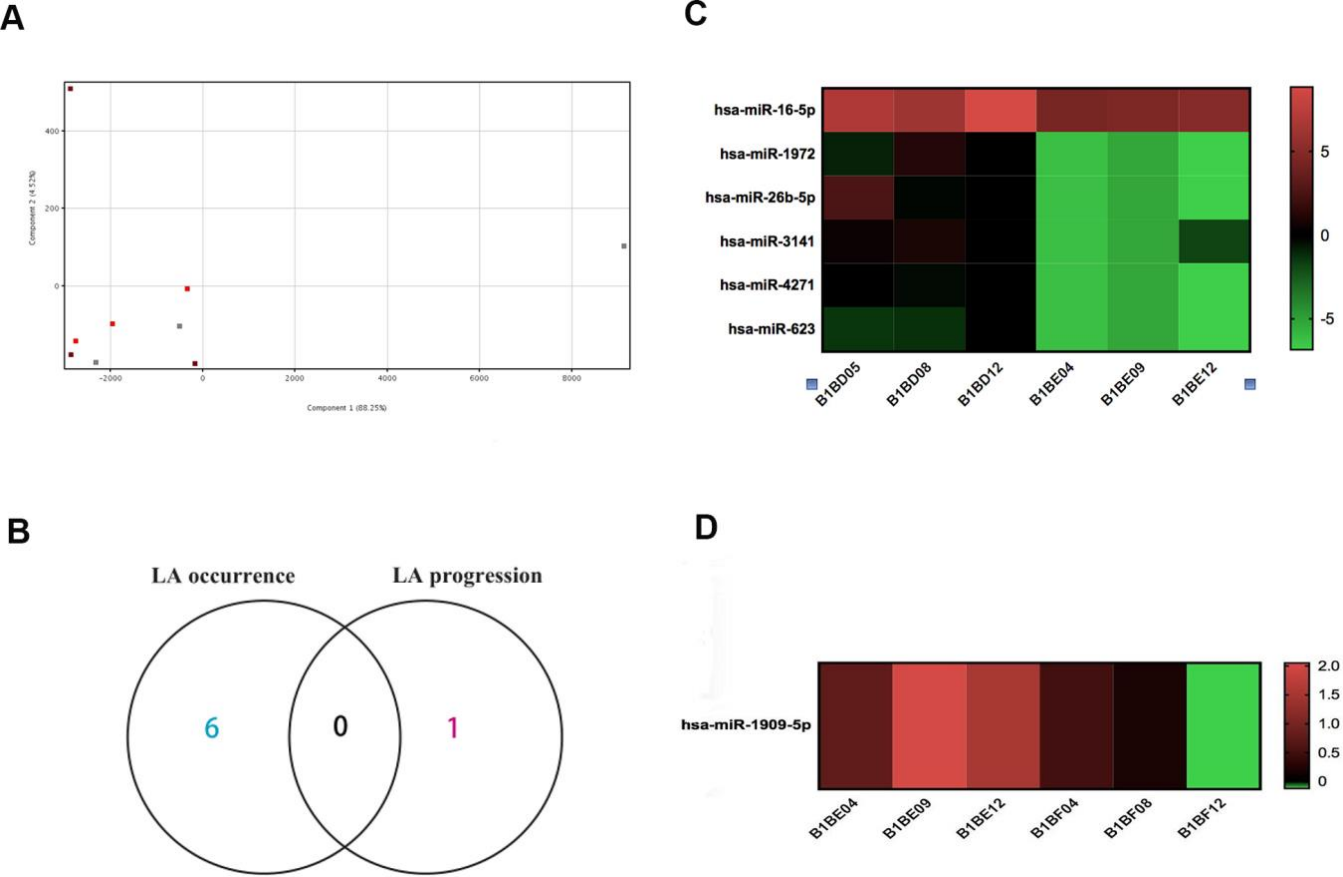
**Microarray miRNA expression profiling of LA in whole blood.** (**A**) PCA of samples shows a small difference among the three groups of subjects. Each symbol represents one subject, with red symbols indicating subjects with type I LA (n=3), orange indicating subjects with type II LA (n=3), and gray indicating subjects without LA (n=3). (**B**) Venn diagram of differentially expressed miRNA identified in the comparison of LA type I vs. Normal and in the comparison of LA type II vs. LA type I did not reveal genes common to onset-associated miRNA and progression-associated miRNA. (**C**) Heatmap of miRNA expression profiling showed six differentially expressed miRNAs identified in the comparison of LA type I (B1BE04, B1BE09, and B1BE12) vs. Normal (B1BD05, B1BD08, and B1BD12). The miRNAs were regarded as LA onset-associated miRNAs. (**D**) Heatmap of miRNA expression profiling showing a single, differentially expressed miRNA in the comparison between LA type II (B1BF04, B1BF08, and B1BF12) vs. LA type I (B1BE04, B1BE09, and B1BE12). This miRNA was designated as an LA progression-associated gene. Red indicates up-regulated expression levels of miRNA, and green indicates down-regulated miRNA expression.

To identify miRNA biomarkers of LA, a miRNA expression profiling analysis was performed on six LA blood samples (including three LA type I and three LA type II) and three healthy control samples using the Agilent Human miRNA Microarray. Principal component analysis (PCA) (Figure 1A) showed only a slight difference in the miRNA expression pattern indicating only a small effect on LA pathogenesis. According to our previous method for defining occurrence-associated and progression-associated genes in methylation microarray analysis of LA [59], we further identified differentially expressed miRNA between Type I LA group and control group as LA onset-specific genes, and revealed differentially expressed miRNA between Type I LA group and Type II LA group as LA development-specific genes. Using a criteria of a twofold change and a P value <0.05, we identified six down-regulated miRNAs (hsa-miR-16-5p, hsa-miR-1972, hsa-miR-26b-5p, hsa-miR-3141, hsa-miR-4271, and hsa-miR-623) and one significantly down-regulated miRNA (hsa-miR-1909-5p) during onset and progression, respectively (Figure 1B and Table 1). With the exception of hsa-miR-16-5p and hsa-miR-26b-5p, five of the miRNAs, including hsa-miR-1972, hsa-miR-3141, hsa-miR-4271, hsa-miR-623, and hsa-miR-1909-5p, have not previously been reported in human diseases. Although the number of differentially expressed miRNA was limited, the identified miRNAs showed novel expression patterns in that all of miRNAs consistently decreased at LA onset and during development (Figure 1C, 1D and Table 1). These findings indicated increased expression of many genes during LA pathogenesis.

**Table 1:** Differentially expressed miRNA in the occurrence and progression of LA.

**Systematic name**	**miRBase accession No**	**Fold change (abs)**	**p value**	**Regulation**	**Comparison**	**Disease**
**hsa-miR-16-5p**	MIMAT0000069	6.0244913	0.03750971	down	LA Type I vs. Normal	LA occurrence
**hsa-miR-1972**	MIMAT0009447	74.32003	0.001073011	down	LA Type I vs. Normal	LA occurrence
**hsa-miR-26b-5p**	MIMAT0000083	116.79823	0.002240083	down	LA Type I vs. Normal	LA occurrence
**hsa-miR-3141**	MIMAT0015010	30.184444	0.022176497	down	LA Type I vs. Normal	LA occurrence
**hsa-miR-4271**	MIMAT0016901	63.976357	0.00021	down	LA Type I vs. Normal	LA occurrence
**hsa-miR-623**	MIMAT0003292	37.15073	0.001256693	down	LA Type I vs. Normal	LA occurrence
**hsa-miR-1909-5p**	MIMAT0007882	2.3847258	0.038300004	down	LA Type II vs. Type I	LA progression

### Confirmation of miRNA microarray data

**Figure 2:**
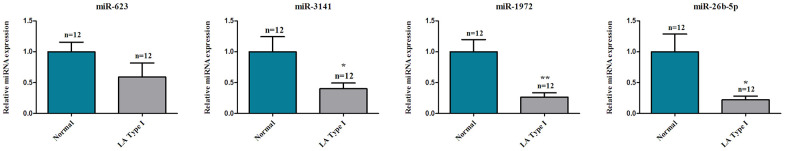
**TaqMan-qPCR analysis of differentially expressed miRNA during LA onset.** Four miRNAs are differentially expressed during LA onset including hsa-miR-1972, hsa-miR-26b-5p, hsa-miR-3141, and hsa-miR-623. They were selected from miRNA microarray analysis data and identified in independent type I LA samples using ABI TaqMan probe-based qPCR technology. The study showed significant down-regulation of hsa-miR-1972, hsa-miR-26b-5p, and hsa-miR-3141 in independent subjects with type I LA (n=12) compared with controls (n=12), ^*^P < 0.05, ^*^P < 0.01.

In order to confirm the miRNA microarray data, we measured the expression of identified miRNAs in an independent cohort of subjects using an ABI TaqMan probe-based, real-time PCR. As the main goal of this work was to identify miRNAs involved in LA pathogenesis, we assayed four LA onset-associated miRNAs, including hsa-miR-623, hsa-miR-26b-5p, hsa-miR-3141, and hsa-miR-1972. They were selected from the differentially expressed miRNAs between type I LA and the control groups. Twelve subjects with type I LA and 12 controls were examined in real-time PCR to quantify miRNAs. Quantitative PCR confirmed the lower expression level of hsa-miR-26b-5p, hsa-miR-3141, and hsa-miR-1972 in plasma in patients with type I LA as compared to subjects without LA, with the exception of hsa-miR-623 (Figure 2). Therefore, the miRNA expression microarray study could be a useful diagnostic tool for molecular changes occurring in the plasma of subjects with LA. Nevertheless, those miRNAs including hsa-miR-26b-5p, hsa-miR-3141, and hsa-miR-1972 may be useful for assessing the early onset of white matter lesions.

### Integrated analysis of miRNA targets databases and mRNA expression profiling of LA

**Figure 3:**
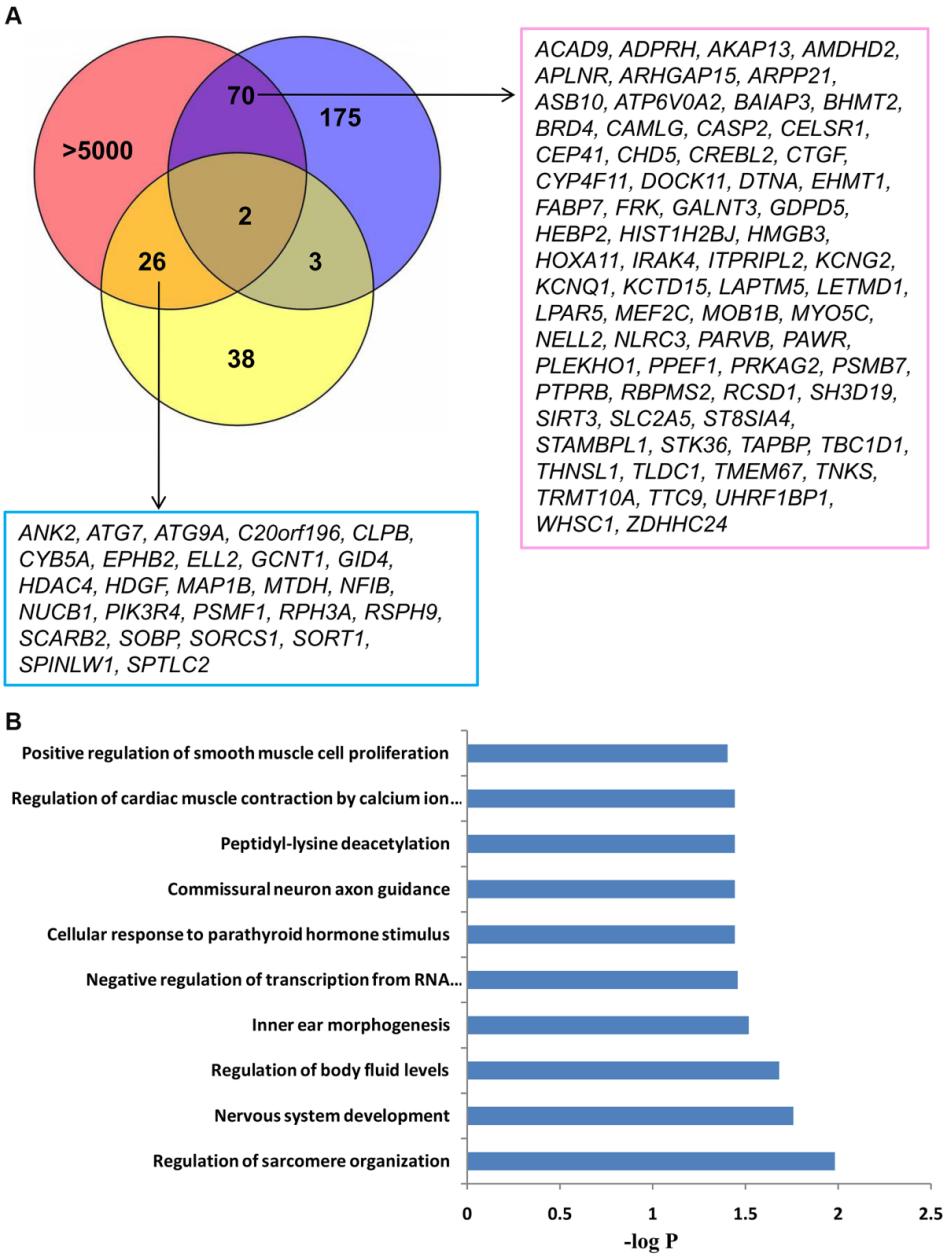
**The identification and GO analysis of up-regulated targets of miRNAs associated with LA.** (**A**) An integrated analysis of microarray LA miRNA and mRNA expression profiling identified 98 up-regulated genes, which could be inhibited by hsa-miR-1972, hsa-miR-26b-5p, or hsa-miR-3141. Two targets showed increased expression in both WML tissue and whole blood of LA subjects. Blue and yellow circles show 250 up-regulated genes in WML tissue and 79 up-regulated genes in whole blood, respectively. The pink circle includes more than 5000 potential targets of miR-1972, miR-3141, and miR-26b-5p, which were predicted from multiple predictive tools. (**B**) Top 10 biological processes significantly enriched with 98 overlapping genes. Functional enrichment analysis was performed using a DAVID bioinformatics database.

In order to screen potential genes targeted by miRNAs implicated in LA and further elucidate their function in the pathogenesis of LA, we took advantage of distinctive gene expression profiling of LA in whole blood and brain tissue and performed an integrated analysis of miRNA and mRNA expression profiling of LA. To identify those significant genes that were inversely correlated with down-regulated miRNAs, we selected 250 up-regulated genes from WML tissue and 69 up-regulated genes from WML blood for association analysis. As shown in Figure 3, more than five thousand genes were predicted by TargetScan 7.1, miRDB, and Pic-Tar software to be targets of hsa-miR-26b-5p, hsa-miR-3141, and hsa-miR-1972. Among these genes, 70 up-regulated mRNAs (such as *ARPP21, CAPS2, DOCK11, FABP7, RCSD1, BAIAP3, KCNQ1, NLRC3*) in WML tissue and 26 up-regulated mRNAs s (such as *CLPB, EPHB1, MTDH, RPH3A, SCARB2, ANK2*) in WML whole blood may be targets of the miRNAs (Figure 3A). In addition, five genes (including *ALAS2, KLHL6, PARVA, PIP5K1B* and *SLC15A2*) were found to overlap in the gene expression profile of white matter lesions taken from brain tissues and whole blood. Among the shared genes, *KLHL6* and *SLC15A2* were predicted to be potential targets of hsa-miR-3141 and hsa-miR-1972, respectively. The over-expression of *KLHL6* and *SLC15A2* in both WML tissue and whole blood of subjects with LA may be attributed to a lower level of inhibition due to the down-regulation of miRNAs affecting the expression of both genes. Taken together, 98 mRNAs, especially *KLHL6* and *SLC15A2,* comprise a set of important genes that may contribute to LA pathogenesis through miRNA-mediated epigenetic mechanisms. Therefore, they were further examined in functional enrichment analyses.

### Functional enrichment analysis of targets of hsa-miR-1972, hsa-miR-3141, and hsa-miR-26b-5p

**Figure 4:**
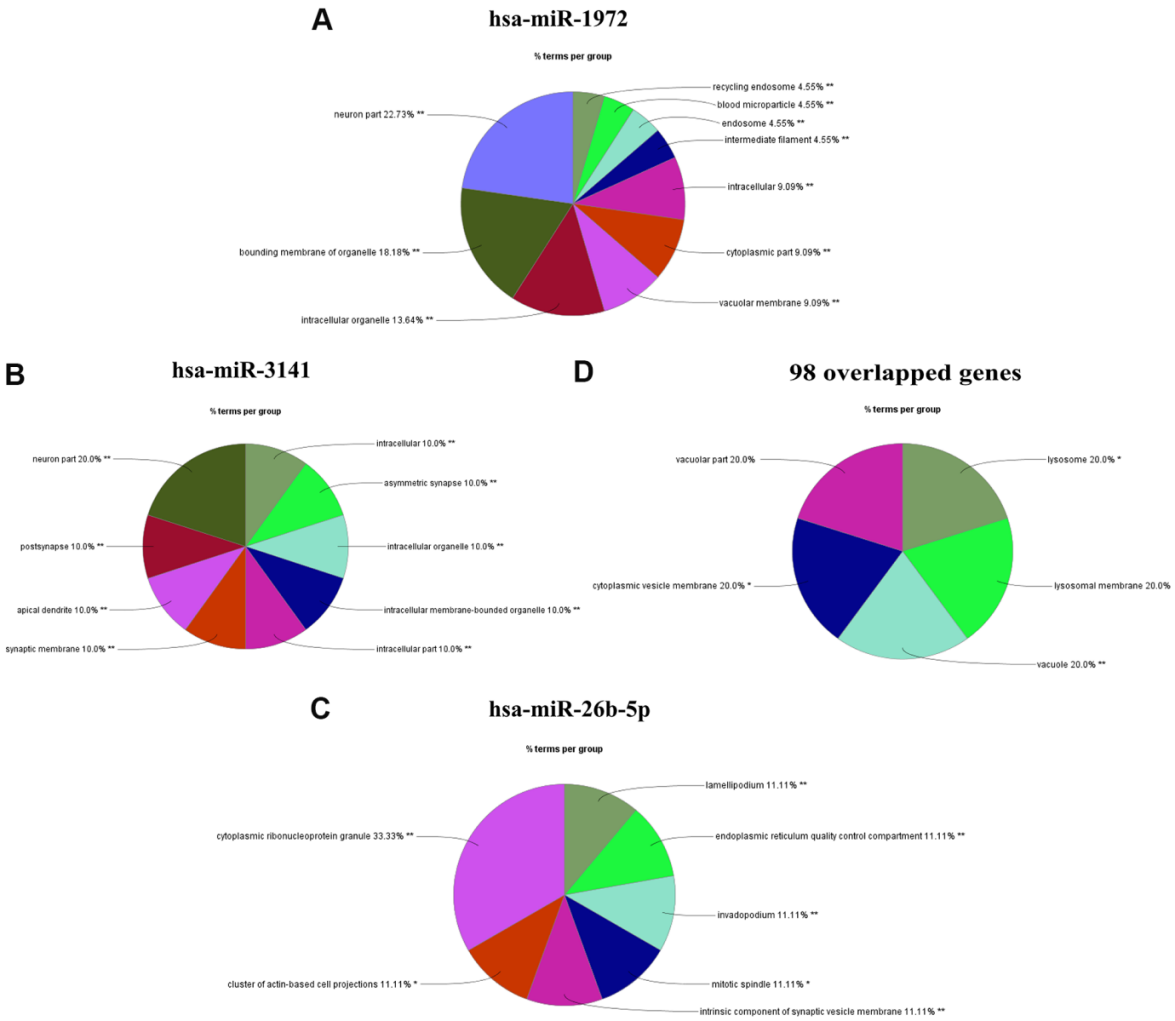
**Functional enrichment analysis of miRNA targets using Cytoscape.** (**A**–**C**) Cellular component enrichment analysis of the predictive targets of miR-1972(**A**), miR-3141(**B**) and miR-26b-5p (**C**). The putative targets of miRNA were predicted by TargetScan 7.1 software. The functional enrichment analysis was performed by the ClueGO tool in Cytoscape software. (**D**). The functional enrichment of the 98 shared genes, shown in Figure 3A. *P < 0.05, **P <0.01.

MiRNA often plays crucial roles in biological processes and human diseases through the direct regulation of mRNA transcription. In order to understand the possible functions of the miRNAs (hsa-miR-1972, hsa-miR-3141, and hsa-miR-26b-5p), we performed a functional enrichment analysis on their predicted targets using the ClueGO tool contained in the Cytoscape software platform. As shown in the Figure 4A, 4B, most of the genes targeted by hsa-miR-1972 and hsa-miR-3141 are mainly enriched in organelle membranes, intracellular organelles, and neuron parts such as synaptic membranes, postsynaptic sites, and dendrites, where they could play important roles in neurogenesis, central nervous system development and differentiation, and regulation of synaptic plasticity (Supplementary Figure 2). These findings strongly indicate specific roles of both miRNAs in the central nervous system. Most targets of hsa-miR-26b-5p are mainly located in cytoplasmic ribonucleoprotein granules, a cluster of actin-based cell projections, mitotic spindles, invadopodia, lamellipodia, and endoplasmic reticulum, where they may positively regulate transcription, cell cycle, and cell or subcellular component movement (Figure 4C and Supplementary 3). Moreover, some of these genes are also enriched in intrinsic components of synaptic vesicle membranes and participate in negatively regulating axonogenesis (Figure 4C and Supplementary Figure 3). These results not only support the significance of its wide distribution in the cell but also implicate additional roles for hsa-miR-26b-5p targets in the nervous system. Additionally, we also conducted GO analysis on all 98 up-regulated targets of hsa-miR-1972, hsa-miR-3141, and hsa-miR-26b-5p, and found that these targets mainly located in the vacuole and vacuolar part, cytoplasmic vesicle membrane, lysosome, and lysosomal membrane (Figure 4D). Their activity was significantly enriched with negative regulation of transcription by the RNA polymerase II, nervous system development and commissural neuron axon guidance (Figure 3B). This bioinformatic evidence strongly supports an important role for these potential targets in the brain. Taken together, we propose that these miRNAs also perform an important role in LA pathogenesis.

### The association of hsa-miR-1972 with *BAIAP3* expression in LA

**Figure 5:**
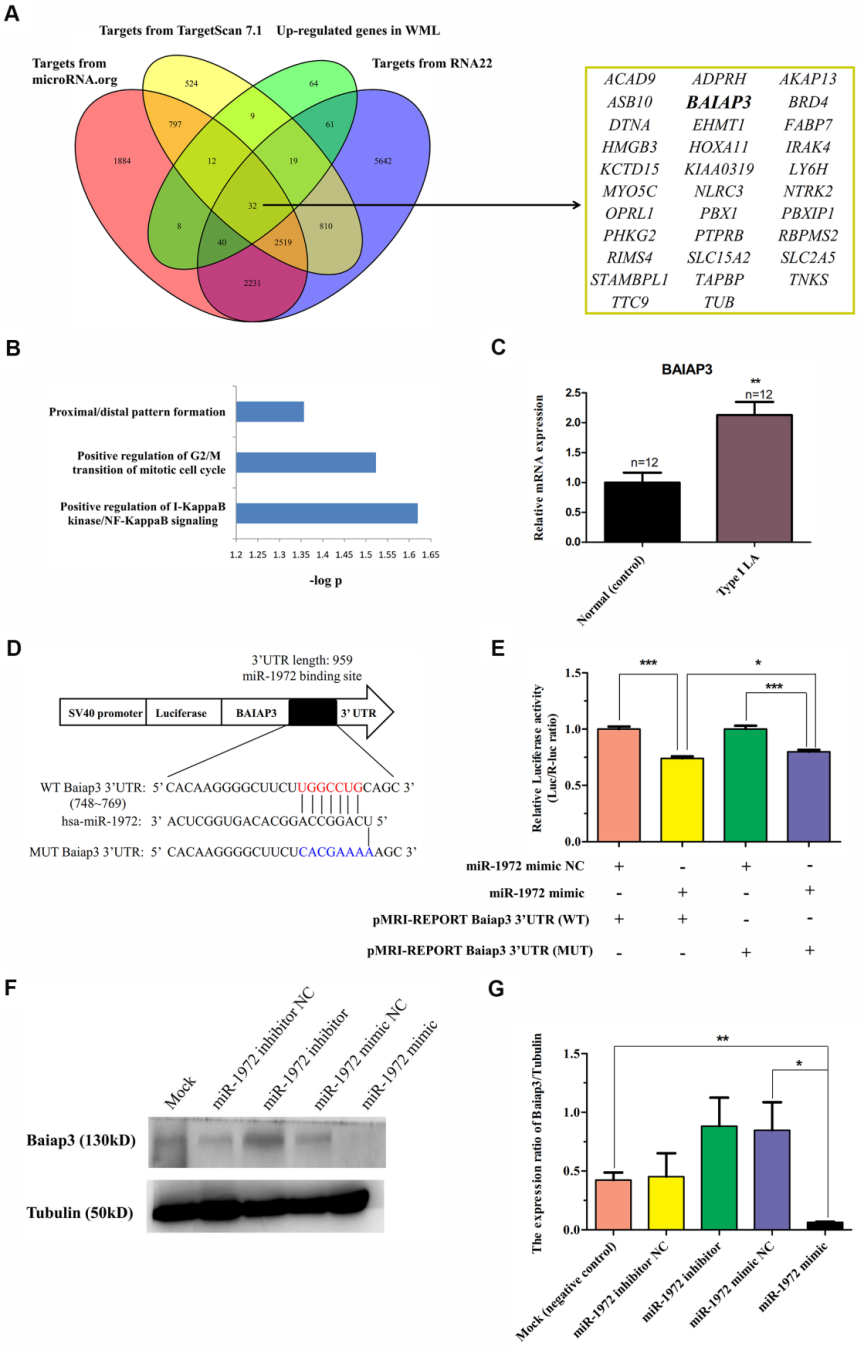
**Identification of BAIAP3 as a target of hsa-miR-1972 in LA.** (**A**) The schematic diagram used to search for targets of hsa-miR-1972 in three databases of miRNA targets that were significantly up-regulated in WML tissues. A total of 32 targets of this miRNAs consistently predicted by three predictive tools showed up-regulation in WML tissues compared with non-lesional white matter samples obtained from subjects with WMLs. (**B**) Functional enrichment analysis of 32 targets of hsa-miR-1972 using DAVID software. Biological processes displaying a statistically significant change with P<0.05 are presented. (**C**) Expression of BAIAP3 in LA patients. The mRNA level of BAIAP3 was determined using real-time PCR in whole blood from type I LA subjects (n=12) and controls (n=12). (**D**) Diagram of predicted miR-1972 binding sites on the 3’UTR of *BAIAP3* gene. The 3’UTR mutant sequence of *BAIAP3* inserted in dual-luciferase reporter genes is indicated in blue. (**E**) Effect of hsa-miR-1972 on *BAIAP3* mRNA transcription in 293T cells using the dual-luciferase reporter assay. Relative luciferase activity was analyzed in 293T cells co-transfected with pMRI-REPORT vector carrying either wild-type or mutated 3’UTR of *BAIAP3* and hsa-miR-1972 mimic or relative negative control. (**F**) Effect of hsa-miR-1972 on the expression level of BAIAP3 protein in 293T cells observed in western blots. 293T cell lines were transfected with miR-1972 mimic (125 nM), miR-1972 inhibitor (125 nM), negative mimic control (125 nM), or negative inhibitor control (125 nM). Protein levels of BAIAP3 were detected at 48 h after post-transfection using western blotting. Tubulin was used as an internal control. (**G**) Quantitative analysis of results obtained from three independent experiments which are presented in (**F**). The results show that hsa-miR-1972 can significantly inhibit the expression of BAIAP3 *in vitro*. ^*^P < 0.05, ^*^P < 0.01, ^*^P < 0.001.

In order to elucidate the manner in which miRNA-mRNA interacted during the pathogenesis of LA, we focused on hsa-miR-1972, which displayed the most significant change in expression, and the up-regulated mRNAs in WML tissue. A total of 32 key genes were identified using multiple predictive tools for miRNA targets, such as TargetScan 7.1, RNA22, and microRNA.org (Figure 5A). They were not only enriched in non-neuronal processes, including positively regulating I-KappaB kinase/NF-KappaB signaling and G2/M transition of mitotic cell cycle and proximal/distal pattern formation (Figure 5B), but were also significantly involved in trans-synaptic signaling by neuropeptide modulation of synaptic transmission (data not shown). Based on the strong enrichment of those genes in vesicle membranes and neural function, we selected *BAIAP3*, a novel gene associated with vesicle exocytosis, for study. As shown in Figure 5C, this gene increased its mRNA expression level in type I LA blood compared to controls. Its overexpression in LA tissue and increased expression *BAIAP3* in LA blood revealed an inverse correlation between *BAIAP3* expression and hsa-miR-1972 expression in LA. It further prompted us to confirm their relationship *in vitro* using a dual-luciferase reporter gene assay and miRNA mimic transfection test. As shown in Figure 5D, wild-type (WT) and mutant-type (MUT) BAIAP3 3’UTR sequences were cloned into dual-luciferase reporter vector. We found that the relative luciferase expression of the BAIAP3-WT + miR-1972 mimic group was significantly decreased compared with the BAIAP3-WT + mimic negative control (NC). However, compared with the BAIAP3-WT expression system, the inhibitory effect of the miR-1972 mimic on relative luciferase expression was significantly lessened in the BAIAP3-MUT expression system (Figure 5E). These results imply that miR-1972 might bind to the bases 762–768 at the 3’UTR site of *BAIAP3* and inhibit BAIAP3 mRNA expression. Unexpectedly, the relative luciferase expression of miR-1972 mimic group did not return to the level of the mimic NC group in the BAIAP3-MUT expression system, indicating the presence of other potential binding sites of miR-1972 located near the 3’UTR site of *BAIAP3* (Figure 5E). Furthermore, we found that the hsa-miR-1972 mimic dramatically decreased the expression of BAIAP3 protein in 293T cell lines. Moreover, the hsa-miR-1972 inhibitor enhanced endogenous BAIAP3 protein levels *in vitro* (Figure 5F, 5G). Collectively, these findings suggest that *BAIAP3* could be targeted and inhibited by hsa-miR-1972 *in vitro*.

## DISCUSSION

Although large-scale genetic studies of European descendants have identified many risk genes and single nucleotide polymorphisms (SNPs) in European descent, a small amount of SNPs from those risk genes has been confirmed in cross-ethnic genetic studies [22, 24, 25, 27, 29–35]. This not only indicates significant ethnic differences in LA susceptibility but also suggests a limited contribution from SNPs to LA pathogenesis. As an important regulatory mechanism, epigenetics has been shown to be critically involved in human disorders, including solid tumors (such as CNS tumor, lung cancer, and breast cancer) [60, 61], and neurodegenerative diseases (such as Parkinson’s disease and Alzheimer’s disease) [62, 63]. Therefore, epigenetic analyses are likely to provide new insights into understanding the pathogenesis of this complex disorder, which may entail both genetic and environmental factors. Indeed, our recent study identified a large number of epigenetic genes that displayed significant changes in DNA methylation at onset and during the progression of LA [59]. Due to its small size and location within body fluids, miRNAs may move from the blood into cerebrospinal fluid or brain tissue during blood-brain barrier (BBB) breakdown and regulate the expression of genes in brain [7, 64–67]. On the contrary, miRNA released from neural cells in the brain could also pass through the BBB and get into the blood, suggesting that the miRNA in the human blood may reflect the disease status in the CNS [68]. Therefore, blood miRNA expression microarray analysis may provide a novel strategy to decipher the molecular mechanisms underlying white matter lesions.

In the present study, we investigated the miRNA expression profile of subjects having LA and identified a limited number of miRNAs that displayed significant changes in expression during disease onset and progression. Among the seven miRNAs identified, four miRNAs that were significantly down-regulated during LA onset were selected for monitoring expression changes in independent participants. We confirmed the significant reduction in their expression levels in subjects with type I LA, which suggested that this set of miRNAs, including hsa-miR-1972, hsa-miR-3141, and hsa-miR-26b-5p, might constitute candidate biomarkers for LA onset. In particular, hsa-miR-1972, was found to target *BAIAP3* and inhibit its expression *in vitro*. Accordingly, we propose that down-regulation of hsa-miR-1972 suppresses the inhibition of *BAIAP3* expression resulting in an increase in *BAIAP3* expression in LA, thereby contributing to LA pathogenesis.

During the past two decades, miRNAs have been shown to be key regulatory factors in cell development and differentiation [69, 70] and to be involved in human diseases [51, 71, 72]. For example, miR-9 and miR-124 drive neuronal differentiation by inhibiting the nuclear receptor TLX, RNA-binding protein PTBP, and transcription factor Sox9 [73–75]. In addition, both miR-219 and miR-338 are also known to promote oligodendrocyte differentiation and myelination by directly targeting and repressing the expression of transcription factors SOX6 and HES5 [76]. Recently, it has been found that the expression levels of miR-135 decrease in the serotonergic raphe nucleus of individuals having major depression disorder (MDD) and that miR-135 targets both the gene encoding the serotonin transporter (SERT) and the gene encoding 5-hydroxytryptamine receptor 1A (HTR1A) *in vitro* in mouse tissue [77]. Another study has also shown that a decrease in the expression of miR-29a or miR-29b increases the expression of BACE1 in brain tissue of subjects with Alzheimer’s disease [78]. Taken together, miRNA can perform a wide variety of functions in various life process and pathological conditions. Understanding the complex roles played by miRNA in pathology and physiology will be helpful for developing efficient diagnostic and treatment strategies for human diseases.

Among the seven miRNAs identified in LA, with the exceptions of hsa-miR-16-5p and hsa-miR-26b-5p, hsa-miR-1972, hsa-miR-3141, hsa-miR-4271, hsa-miR-623, and hsa-miR-1909-5p have for the first time been reported to be involved with disease. They may have many unknown functions in the central nervous system. To further explore the roles of these prospective miRNAs, we used *in silico* predictions of miRNA targets combined with integrated analysis of miRNA and mRNA expression profiling to identify likely targets. Gene Ontology enrichment analysis was also conducted to determine the functional classification of identified genes. Consistent with the functional enrichment analysis results of all predictive targets of hsa-miR-1972 or hsa-miR-3141, 98 up-regulated targets of hsa-miR-26b-5p, hsa-miR-1972, and hsa-miR-3141 in white matter lesions tissues were also enriched during nervous system development (such as *MEF2C, HDAC4, ST8SIA4, MAP1B, FABP7,* and *EPHB2*) and neurogenesis (such as *NFIB* and *EPHB2*). Several of the targeted genes have also been reported in cerebral cortex neuronal differentiation (such as GDPD5 and CHD5), inflammatory and immune response pathways including I-kB kinase/NF-kB signaling (such as *IRAK4, MTDH, AKAP13,* and *BRD4*), toll-like receptor 9 signaling (such as *IRAK4* and *PIK3R4*), and antigen processing and presentation of exogenous peptide antigen via MHC class I (such as *PSMB7, PSMF1,* and *TAPBP*). This bioinformatics information is a strong indication that their potential mechanisms contribute to the underlying cerebral white matter hyperintensities in CNS. Since LA is a demyelinating disorder that occurs in cerebral white matter, LA may not be solely due to neuroinflammation within central nervous system [23, 58, 59, 65, 79]. The inability for nervous system differentiation (such as oligodendrocyte differentiation) and neurogenesis (such as neuron axonogenesis) to occur may also contribute to the abnormal remyelination occurring on neuronal axons in white matter.

We also found in our miRNA-mRNA interaction studies that the expression of has-miR-1972 was significantly (P<0.01), inversely correlated with the expression of *BAIAP3*, which was up-regulated both in whole blood and the white matter lesions of LA subjects. *BAIAP3* has recently been identified to be a novel member of the cell-specific Munc13 family. It encodes the brain-specific angiogenesis inhibitor I-associated protein 3 (Baiap3), which is expressed in the amygdala, hypothalamus, and periaqueductal gray regions of the brain [80, 81]. As with other Munc13 proteins, BAIAP3 is also involved in the regulation of secretory pathways [81]. Recently, it has been shown to indirectly activate dense-core vesicle (DCV) exocytosis by promoting DCV maturation through its positive role in retrograde trafficking of DCV proteins to the trans-Golgi network (TGN) in neuroendocrine cells [82, 83]. Neurotrophins, such as brain-derived neurotrophic factor (BDNF), have been shown to play critical roles in the cell growth, survival, and differentiation of neurons. They are released from secretory vesicles and are involved in the regulation of synaptic plasticity, adult neurogenesis, and normal brain function as well as neuronal and psychiatric diseases [84–86]. The secretory granule-associated protein CAPS2 has been reported to promote neurotrophin BDNF release and cell survival in Purkinje cells [84]. Its paralog CAPS1 has been shown to enhance DCV trafficking and increase the probability of presynaptic release, thus controlling synaptic transmission in the mouse brain [87]. Therefore, we hypothesize that aberrant *BAIAP3* inhibited by hsa-miR-1972 may contribute to the pathogenesis of white matter lesions by affecting neuronal survival, differentiation, and neurogenesis by indirectly regulating DCV trafficking, exocytosis, and subsequent molecular signaling. These potential functions of BAIAP3 in neurogenesis and remyelination via DCV exocytosis will require investigation *in vitro* and *vivo*.

To our knowledge, this is the first study to characterize the miRNA profiling of LA. It identifies a set of aberrant miRNAs—has-miR-1972, hsa-miR-3141, hsa-miR-26b-5p and hsa-miR-1909-5p—implicated in the onset and progression of LA. These miRNAs may be useful biomarkers for monitoring the development of LA. In addition, they may also help elucidate the epigenetic mechanism underlying white matter lesions. However, this study has some limitations: 1) Small sample size may lead to false positive results. Future studies with larger sample size are required to replicate the identification of candidate miRNA biomarkers for LA. 2) Low sample quality due to the total RNA extraction from the frozen whole blood resulted in the observation of relatively small amount of significant miRNAs. 3) The absence of time points did not permit monitoring time-course of molecular events during the development of LA. Notably, the transcriptome of this complex disorder is likely to change throughout an individual’s lifetime. Therefore, this study only provides one portrait of LA miRNA expression profiling at a single point in time. In future studies, it will be necessary to determine miRNA profiling during development at multiple time points. 4) The miRNAs were not isolated from brain tissue but instead blood samples of LA patients. Although gene expression changes in whole blood are considered to mirror those in the brain, miRNA profiling of whole blood is unlikely to reflect the full range of molecular events in the brain. 5) The precise roles of has-miR-1972 and BAIAP3 in the demyelination pathology of LA have not been studied in an animal model. Therefore, further studies are needed to elucidate the putative mechanisms of LA involving miRNA and its associated targets.

## CONCLUSIONS

This study is the first to characterize miRNA expression profiling of LA. We identified significantly dysregulated miRNAs in the onset and progression of LA, and revealed a large number of targets of those miRNAs through integrated analysis of miRNA and mRNA expression profiling of LA. *BAIAP3* was confirmed as a novel target of hsa-miR-1972 *in vitro*. Functional enrichment analyses of those miRNAs targets further highlighted pathogenesis during neurogenesis, neural development, and neural differentiation. Our findings provide insight concerning the epigenetic mechanisms involved in LA onset and development. They may also have clinical implications for diagnosis in the monitoring of LA.

## MATERIALS AND METHODS

### Study subjects

Whole blood and plasma samples were collected from nine typical subjects. As shown in Supplementary Figure 1, they were divided into three subgroups: normal (controls without LA), LA Type I, and LA Type II according to the definition and classification scheme of LA previously described in our published papers [23, 59]. The classification was based on the severity of WMLs. It did not include the neuroimaging distinctions between periventricular WMLs and deep/subcortical WMLs and differentiated between early onset LA and severe LA. Thus, the six subjects with LA included in the present study represented two different pathological states of cerebral white matter. All of the subjects were aged from 60 to 80 years and were free of hypertension, diabetes, neurologic disorders (such as dementia, Parkinson’ disease, Alzheimer’s disease, stroke, multiple sclerosis, and hydrocephalus), psychiatric disorders (such as depression and schizophrenia) and inflammatory diseases (such as systemic lupus erythematous), brain trauma, intracerebral hemorrhage, subarachnoid hemorrhage, intracranial infection, malignant tumor, ischemic heart disease, and toxic encephalopathy, as well as other severe diseases. Clinical details of these subjects are shown in Supplementary Table 1. In order to confirm miRNA expression microarray data and detect gene expression of the miRNA targets, we further recruited 24 independent subjects, which included 12 subjects with type I LA and 12 healthy controls from the Department of Neurology of the First Affiliated Hospital of Xiamen University. All subjects included in this study provided written informed consent. This study was approved by the Xiamen University Ethical Committee.

### Total RNA extraction

Total RNA from nine whole blood samples was extracted using MagCore whole blood RNA extraction kits. The quality and integrity of RNA was determined by means of the NanoDrop®ND-1000 UV–Vis Spectrophotometer (NanoDrop Technologies, Wilmington, USA) and RNA 6000 NanoLabChip Kit (Agilent Technologies), respectively. Total RNA from independent subjects and 293T cell line samples for miRNA and mRNA expression detection were extracted using the TRIZOL method. All of the free total RNA was stored at -80° C for further analysis.

### miRNA microarray data analysis

MiRNA was further labeled with Cy3 using the miRNA Complete Labeling and Hyb Kit (Agilent Technologies). Labeled miRNA was then hybridized using the Agilent Human miRNA 8×60k v.18.0 arrays (Agilent Technologies, Santa Clara, CA, USA). Fluorescent signal intensities were detected and extracted with an Agilent Microarray Scanner (Agilent Technologies) and Feature Extraction 10.7.3.1 Software (Agilent Technologies), respectively. The miRNA array data were further analyzed for data summarization, normalization, and quality control using the GeneSpring software v.12.0 (Agilent Technologies, Inc.). In order to identify expression patterns, we performed a PCA using GeneSpring software before conducting the differential expression analysis. The comparative analysis on miRNA expression level between the LA and control groups was statistically determined using a t-test (P-values). One miRNA with a fold change (FC)>2.0 and P<0.05 was considered to be statistically significant. Results were visualized by means of a heat-map using the genesis program (http://genome.tugraz.at).

### TaqMan-PCR for miRNA quantitation

Mature miRNA in plasma was reverse-transcribed using TaqMan MicroRNA Reverse Transcription Kit (cat no: 4366596, life technology, USA) following the recommended protocol. MiRNAs were then quantified using TaqMan^®^ MicroRNA Assays (cat no: 4427975, cat no: 442795, cat no: 4427975, and cat no: 4440886, Life Technology, USA). All qPCR reactions were performed on StepOne real-time reverse transcription (RT)-PCR (Life Technologies, USA) in triplicate for each sample. The fold changes of miRNA were normalized to a reference, synthetic small RNA, cel-miR-39, which was provided in the TaqMan® MicroRNA Assays (Small-scale, Inventoried)-Assay cel-miR-39 (cat no: 4427975, Life Technology, USA). Finally, miRNA expression level was calculated using the 2^−ΔΔCT^ method.

### Prediction of miRNA targets

MiRNA targets were predicted by means of several independent algorithms or databases, including TargetScan 7.1 (http://www.targetscan.org/vert_71/), miRDB (http://mirdb.org/), PicTar (http://pictar.mdc-berlin.de/), RNA22 (https://cm.jefferson.edu/rna22/Interactive/), microRNA.org, or miRBase (http://mirbase.org/index.shtml). The potential miRNA-mRNA interactions were then obtained. Candidate genes consistently retrieved by three prediction tools, were selected for assessing the relationship between mRNA and miRNA expression and miRNA-mediated regulation of protein expression in clinical and *in vitro* samples, respectively.

### Real-time PCR for mRNA quantitation

First-strand cDNAs were synthesized from 1 μg of total RNA using a M-MLV Reverse Transcriptase Kit (Invitrogen, USA). All qPCR reactions were performed as described in the SYBR Green-based method using the Agilent Mx3005P Real-Time PCR system (Agilent, USA). *GAPDH* was employed as an endogenous control for normalization of qPCR data according to the ΔΔCT method. All qPCR experiments were done in triplicate. Primer pairs were designed using the Primer Premier 5 software (Premier Biosoft International, Palo Alto, CA, USA) and validated by iPAGE.

### Functional enrichment analysis of genes targeted by miRNA

As one open-source software, Cytoscape is often used to analyze and visualize complex, molecular interaction networks, and biological pathways. ClueGO is a Cytoscape plug-in that visualizes the non-redundant biological terms for large gene clusters in functionally grouped networks (http://apps.cytoscape.org/apps/cluego). We used the Clue GO version 2.5.1 contained in the Cytoscape software version 3.6.1 together with a DAVID 6.8 bioinformatics database to perform molecular function and biological process analyses on genes targeted by miRNA. The indicated species was Homo sapiens, and the Benjamini–Hochberg method was used to obtain P values for multiple tests. An adjusted P-value of < 0.05 was considered statistically significant.

### Dual-luciferase reporter gene assay

The dual-luciferase reporter assay system (E1910, Promega) was used to determine whether *BAIAP3* was a direct target of hsa-miR-1972. First, the wild-type 3’UTR sequences of Baiap3 mRNA having a full length of 959 nucleotides, which included putative binding sites for hsa-miR-1972 (5’CACAAGGGGCUUCUUGGCCUGC3’ [position:748–769]) and corresponding mutated sites (5’ CACAAGGGGCUUCUCACGAAAA 3’) were cloned into the pMIR-REPORT dual-luciferase reporter vector (Reported fluorescence for hluc, corrected fluorescence for hRluc as the internal reference). The reconstituted plasmids were named as pMRI-REPORT BAIAP3 3’UTR-WT and pMRI-REPORT BAIAP3 3’UTR-MUT, respectively. The 293T cells were seeded in 96-well plates at a density of 5×10^3^ cells/mL for 24 h and then co-transfected with hsa-miR-1972 mimics or negative controls (NC) at a final concentration of 100 nM, with 0.2 μg/well of pMRI-REPORT BAIAP3 3’UTR-WT or pMRI-REPORT BAIAP3 3’UTR-MUT and 0.004 μg/well of Renilla luciferase plasmids for 48 h using lipofectamine 2000 (11668-019, Invitrogen). Firefly and Renilla luciferase activities were measured using the Dual-Luciferase Reporter Assay method. Renilla luciferase activity was then used for normalization of luciferase activity to minimize differences in transfection efficiency. The ratio of firefly fluorescence to Renilla fluorescence was designated as the relative luciferase activity. All experiments were performed at least three times.

### Cell culture and cell transfection of miRNA mimic and inhibitor

Human 293T cell lines were cultured in DMEM/F12 medium (HycloneTM, Thermo) supplemented with 10% fetal bovine serum (FBS) (Gibco, USA), 100 units/mL penicillin, and 100 μg/mL streptomycin at 37° C in a 5%CO2 incubator. When cells seeded in 6-well plates reached a level of 40% confluence, 293T cells were transfected with miRNA mimic (has-miR-1972 mimic: cat no: miR10009447, RiboBio, Guangzhou, China) and miRNA inhibitor (has-miR-1972 inhibitor: cat no: miR20009447, RiboBio, Guangzhou, China) by Lipofectamine2000 (Invitrogen, Carlsbad, CA, USA) according to the manufacturer’s protocol. Proteins were extracted for analysis 48 h after miRNA mimic was added. All experiments were performed at least three times.

### Western blot

Total cell extracts were harvested in radioimmunoprecipitation (RIPA) lysis buffer (Cat. No: R0010-100, Solarbio, China) supplemented with protease inhibitors on ice. Protein quantification was determined with a PierceTM BCA protein assay kit (Cat. No: 23227, Thermo Fisher Scientific, Waltham, MA, USA). Protein detection was performed using SDS-PAGE, and western blots were carried out according to standard methods. Proteins were separated by 10% SDS-PAGE and transferred onto PVDF membranes (Millipore Corporation, Billerica, MA, USA). The membranes were then blocked overnight with 5% non-fat dried milk for 2 h and incubated with anti-BAIAP3 antibody (Cat. No: 24836-1-AP, Proteintech, USA) at 1:5000 dilution, and anti-Tubulin antibody (Cat. No: 11224-1-AP, Proteintech, USA) at 1:50,000 dilutions overnight at 4° C. After washing with TBST (10 mM Tris, pH 8.0, 150 mM NaCl, and 0.1% Tween20), the membranes were incubated for 4 h at room temperature with goat anti-rabbit second antibody at 1:20,000. Finally, the protein bands were detected by enhanced chemiluminescence (ECL) (Advansta, USA) with a BioSpectrum Gel Imaging System (UVP, USA). All experiments were performed at least three times.

### Statistical analysis

Data on the miRNA and mRNA expression levels are reported as means ± SEM. An unpaired t test was used to analyze the statistical significance of miRNA and mRNA expression. All P values were calculated using a two-sided test, and a P value of less than 0.05 (P <0.05) was considered statistically significant. All statistical analyses and graph constructions were performed using the SPSS software version 17.0 (IBM, USA) and GraphPad Prism Software version 5.0 (GraphPad, USA).

### Ethical statement and patient consent

This study was approved by Xiamen University ethical committee (2020GKJ041), and it was registered in the Chinese clinical trial registry center (Number: ChiCTR-COC-15007640). All of study subjects gave written informed consent.

## Supplementary Material

Supplementary Figures

Supplementary Table 1
