# Correction for: Integrated analysis of microRNA and mRNA expression profiling identifies BAIAP3 as a novel target of dysregulated hsa-miR-1972 in age-related white matter lesions

**DOI:** 10.18632/aging.203217

**Published:** 2021-06-14

**Authors:** Wen-Qing Huang, Qing Lin, Shuai Chen, Lixiang Sun, Qingjie Chen, Kehui Yi, Zhi Li, Qilin Ma, Chi-Meng Tzeng

**Affiliations:** 1 Shanghai Institute of Precision Medicine (SHIPM), Ninth People’s Hospital, Shanghai Jiao Tong University School of Medicine, Shanghai, China; 2 Translational Medicine Research Center (TMRC), School of Pharmaceutical Sciences, Xiamen University, Xiamen, Fujian, China; 3 Department of Neurology, The First Affiliated Hospital of Xiamen University, Xiamen, Fujian, China; 4 Xiamen Key Laboratory of Brain Center, The First Affiliated Hospital of Xiamen University, Xiamen, Fujian, China; 5 School of Medicine, Xiamen University, Xiamen, Fujian, China; 6 The First Clinical College of Fujian Medical University, Fuzhou, Fujian, China; 7 Department of Otolaryngology-Head and Neck Surgery, Xiamen Key Laboratory of Otolaryngology-Head and Neck Surgery, The First Affiliated Hospital of Xiamen University, Xiamen, Fujian, China; 8 Chen Zhi-nan Academician Workstation, Institute of Basic and Translational Medicine, Xi’an Medical University, Xi’an, Shanxi, China; 9 Department of Nuclear Medicine, The First Affiliated Hospital of Nanchang University, Nanchang, Jiangxi, China; 10 Department of Neurology, Zhongshan Xiamen Hospital, Fudan University, Xiamen, Fujian, China; 11 College of Pharmaceutical Sciences, Nanjing Tech University, Nanjing, Jiangsu, China

**Keywords:** correction

Original article: Aging. 2021; 13:4674–4695.  . https://doi.org/10.18632/aging.202562

**This article has been corrected:** The authors requested to remove the first and last affiliations. This correction does not change the content of the publication. The correct list of authors and affiliations is provided below:

Wen-Qing Huang^1,*^, Qing Lin^2,3,4,5,*^, Shuai Chen^6,7,*^, Lixiang Sun^1,*^, Qingjie Chen^8^, Kehui Yi^2,9^, Zhi Li^1^, Qilin Ma^2,3,4,5,&^, Chi-Meng Tzeng^1^

^1^Translational Medicine Research Center (TMRC), School of Pharmaceutical Sciences, Xiamen University, Xiamen, Fujian, China

^2^Department of Neurology, The First Affiliated Hospital of Xiamen University, Xiamen, Fujian, China

^3^Xiamen Key Laboratory of Brain Center, The First Affiliated Hospital of Xiamen University, Xiamen, Fujian, China

^4^School of Medicine, Xiamen University, Xiamen, Fujian, China

^5^The First Clinical College of Fujian Medical University, Fuzhou, Fujian, China

^6^Department of Otolaryngology-Head and Neck Surgery, Xiamen Key Laboratory of Otolaryngology-Head and Neck Surgery, The First Affiliated Hospital of Xiamen University, Xiamen, Fujian, China

^7^Chen Zhi-nan Academician Workstation, Institute of Basic and Translational Medicine, Xi’an Medical University, Xi’an, Shanxi, China

^8^Department of Nuclear Medicine, The First Affiliated Hospital of Nanchang University, Nanchang, Jiangxi, China

^9^Department of Neurology, Zhongshan Xiamen Hospital, Fudan University, Xiamen, Fujian, China

*Equal contribution

