# Supplementary material for: Integrated analysis of microRNA and mRNA expression profiling identifies BAIAP3 as a novel target of dysregulated hsa-miR-1972 in age-related white matter lesions
Source: Aging (Albany NY). 2021 Feb 9;13(3):4674–95. doi: 10.18632/aging.202562 (PMC7906144; doi:10.18632/aging.202562)
Supplement: Supplementary Figures [file aging-13-202562-s001.pdf]

SUPPLEMENTARY FIGURES

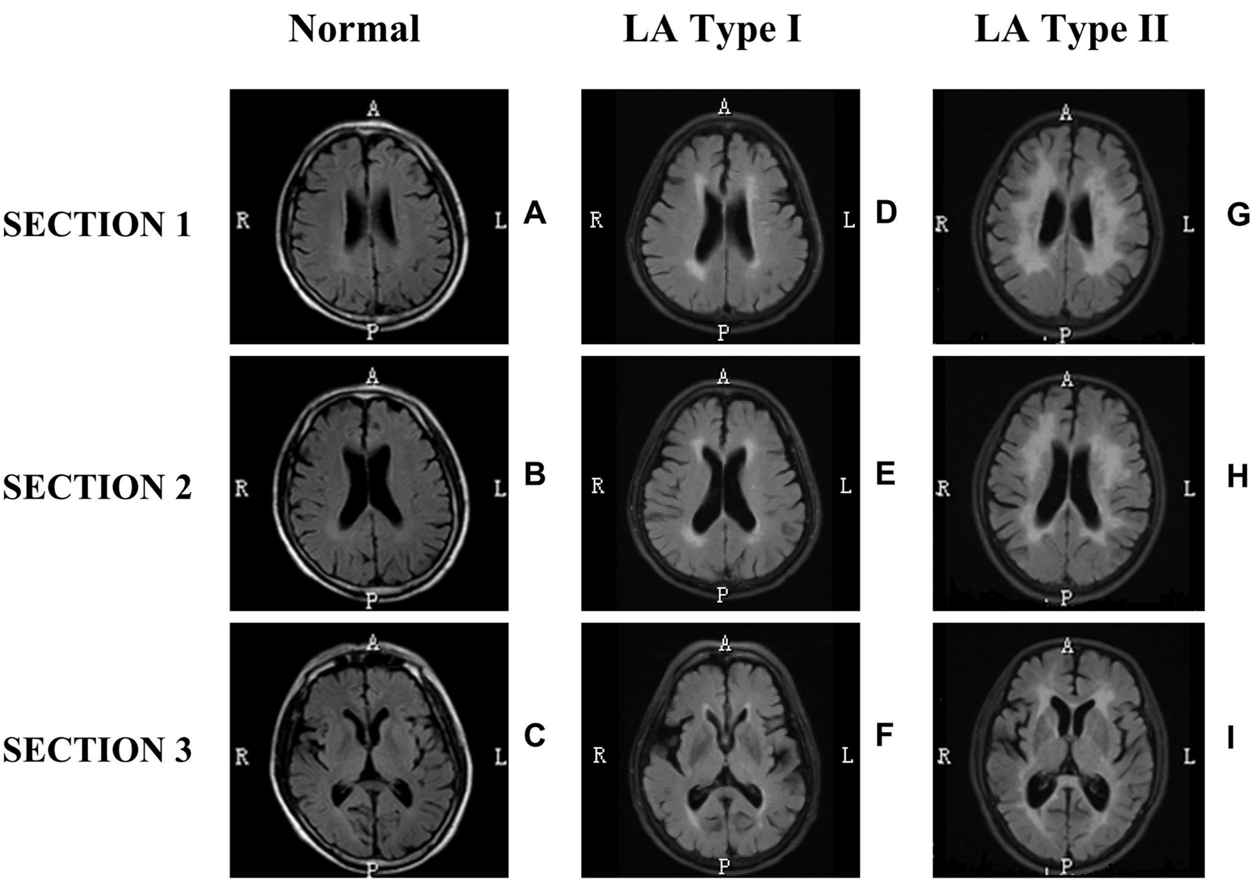

Supplementary Figure 1. Neuroimaging-based classification of subjects. Subjects were divided into three subgroups according to the systematical assessment of white matter lesions on three sections (Sections 1: centrum semiovale section, Section 2: lateral ventricles section, Section 3: internal capsule section) of brain MR imaging. Normal (**A**, **B**, **C**) showing few white matter lesions on all three sections of FLAIR-MRI imaging was considered as control group. LA type I (**D**, **E**, **F**) and LA type II (**G**, **H**, **I**) represent two different severity of white matter lesions on those three sections of T2-weighted FLAIR-MRI scans. This figure was cited from our published paper (Huang et al, Front Aging Neurosci. 2018; 10:143. https://doi.org/10.3389/fnagi.2018.00143).

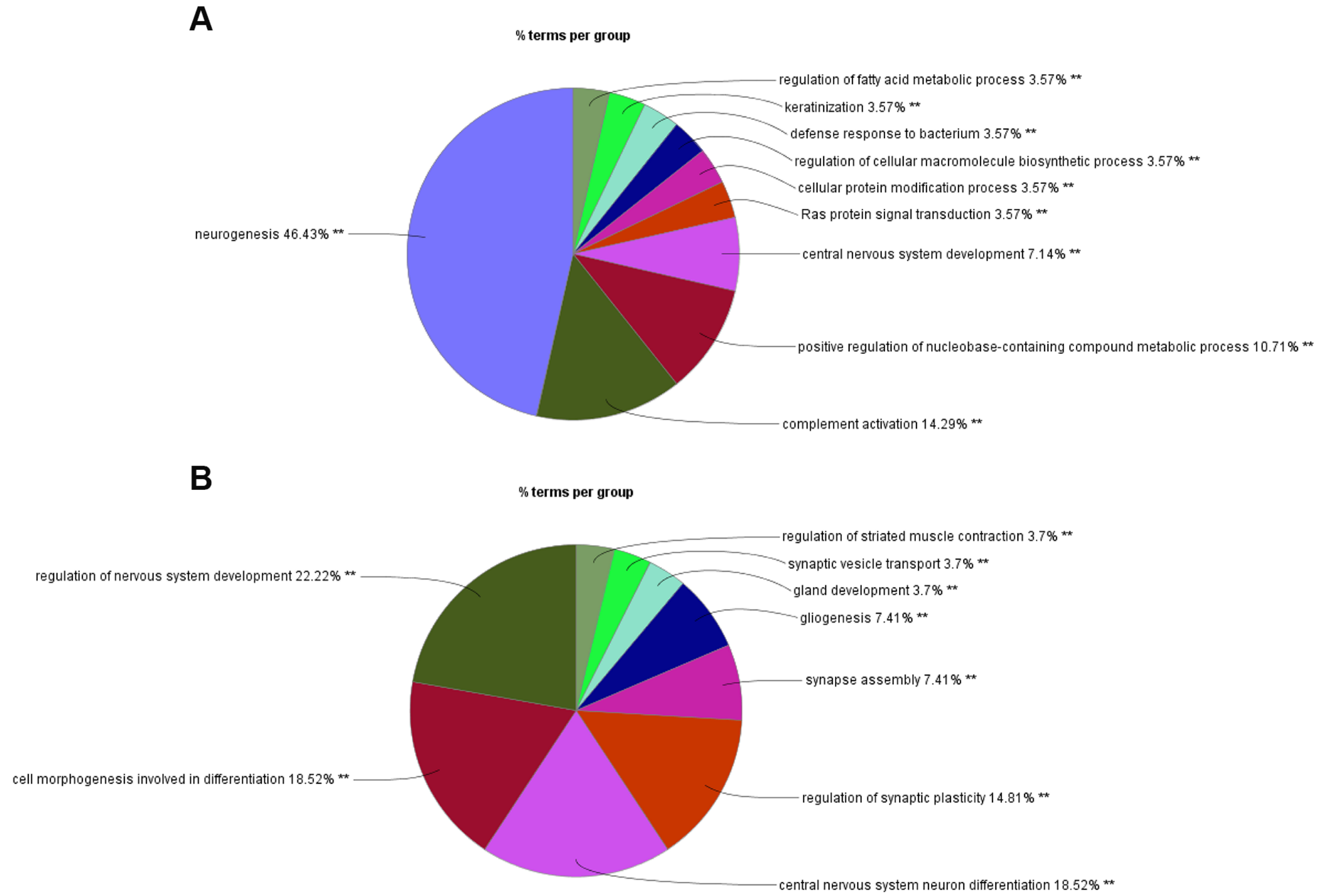

Supplementary Figure 2. Biological processes enrichment analysis of those miRNA targets by Cytoscape. (**A**, **B**) show GO-biological processes analysis on the predictive targets of miR-1972 and miR-3141 by the ClueGO tool contained in Cytoscape software, respectively. The potential targets of both miRNAs were predicted by TargetScan 7.1 software. $^{*}P < 0.05$, $^{**}P < 0.01$.

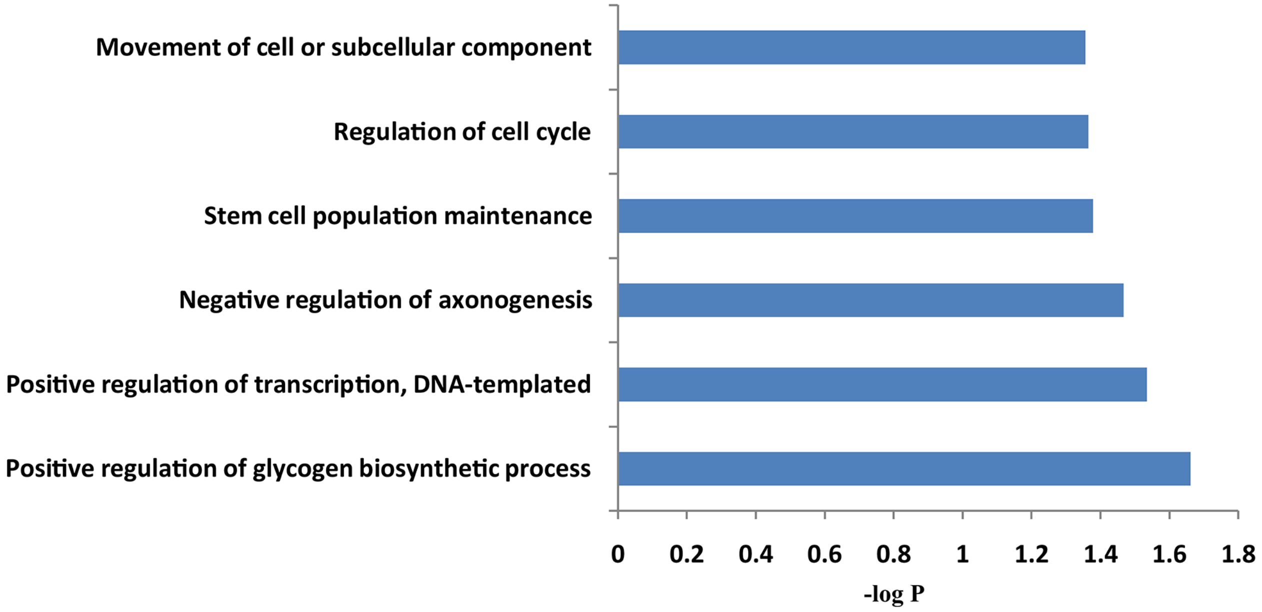

Supplementary Figure 3. Significant biological processes enriched with the predicted targets of miR-26b-5p. Biological processes enrichment analysis was performed by DAVID. The potential targets of miR-26b-5p were predicted by TargetScan 7.1 software.
