# Supplementary material for: Integrated analysis of microRNA and mRNA expression profiling identifies BAIAP3 as a novel target of dysregulated hsa-miR-1972 in age-related white matter lesions
Source: Aging (Albany NY). 2021 Feb 9;13(3):4674–95. doi: 10.18632/aging.202562 (PMC7906144; doi:10.18632/aging.202562)
Supplement: Supplementary Table 1 [file aging-13-202562-s002.pdf]

SUPPLEMENTARY TABLE

Supplementary Table 1. Clinical details of the subjects used in the miRNA microarray study.

Subjects	Label	Age (years old)	Hypertension	Diabetes	Smoking	Drinking	LDL-C (mmol/L)	HCY (μmol/L)	TG (mmol/L)	HCRP (mg/L)	CRE (umol/L)	UA (umol/L)	APOA1 (g/L)	ApoB (g/L)	MRI
1	**B1BD05**	70-75	-	-	-	-	3.14	8.55	0.65	2.7	79	325	1.18	0.89	Normal
2	**B1BD08**	70-75	-	-	-	-	3.65	18.93	1.38	3.0	95	303	1.20	0.95	
3	**B1BD12**	70-75	-	-	+	-	2.95	14.77	1.18	4.2	107	273	1.00	0.74	
4	**B1BE04**	60-65	-	-	+	-	2.89	10.29	0.65	10.9	100	309	0.80	1.26	LA Type I
5	**B1BE09**	60-65	-	-	+	-	1.83	19.61	0.65	12.3	99	237	1.18	0.55	
6	**B1BE12**	70-75	-	-	-	-	3.95	37.93	0.94	2.1	102	291	1.08	0.91	
7	**B1BF04**	60-65	-	-	+	+	3.13	14.81	1.28	6.1	90	283	1.09	0.87	LA Type II
8	**B1BF08**	66-70	-	-	-	-	2.15	13.17	0.95	20.1	79	299	0.76	0.73	
9	**B1BF12**	80-85	-	-	-	-	2.98	26.95	0.88	4.8	101	322	1.10	0.96	

Note: LDL-C: low density lipoprotein cholesterol (range of normal level: 2.07~3.36 mmol/L); HCY: homocysteine (range of normal level: 0~15μmol/L); TG: Triglyceride (range of normal level: 0.5~1.7 mmol/L); HCRP: high-sensitivity C-reactive protein (range of normal level: 0~5 mg/L); CRE: Creatinine (range of normal level: 44~133 umol/L); UA: uric acid (range of normal level: 88~430 umol/L); ApoA1: Apolipoprotein A1(range of normal level: 1.1~1.6 g/L); ApoB: Apolipoprotein B (range of normal level: 0.6~1.1 g/L). LA: leukoaraiosis. Partial information of the supplemental table was cited from our published paper (Huang et al, Front Aging Neurosci. 2018; 10:143. https://doi.org/10.3389/fnagi.2018.00143).
